# Effects of Oral Administration of *Lepidium meyenii* on Morphology of Mice Testis and Motility of Epididymal Sperm Cells After Tetrahydrocannabinol Exposure

**DOI:** 10.3389/fvets.2021.692874

**Published:** 2021-12-09

**Authors:** Adelaide Greco, Chiara Del Prete, Davide De Biase, Veronica Palumbo, Sandra Albanese, Dario Bruzzese, Domenico Carotenuto, Francesca Ciani, Simona Tafuri, Leonardo Meomartino, Marcello Mancini, Orlando Paciello, Natascia Cocchia

**Affiliations:** ^1^Interdepartmental Center of Veterinary Radiology, University of Naples Federico II, Naples, Italy; ^2^Institute of Biostructures and Bioimaging of the National Council of Research, Naples, Italy; ^3^Department of Veterinary Medicine and Animal Production, University of Naples Federico II, Naples, Italy; ^4^Department of Pharmacy, University of Salerno, Fisciano, Italy; ^5^Department of Public Sanity, University of Naples Federico II, Naples, Italy; ^6^Universidad Nacional Mayor San Marcos, Lima, Peru

**Keywords:** *Lepidium meyenii* (maca), sperm cells, THC, antioxidant, ultrasound color Doppler

## Abstract

**Background:** Tetrahydrocannabinol (THC) administration is associated with testicular damage and reduced semen quality. Oral administration of *Lepidium Meyenii* (maca) improves spermatogenesis and sperm motility and count and reduces spermatogenic damage.

**Objectives:** The aim of this study was to evaluate the effect of administration of THC, maca, and their combination on testicular tissue and semen parameters.

**Materials and Methods:** Thirty-six-week-old male mice were classified into control, THC, Maca, and THC + Maca groups. The mice were subjected to Eco Color Doppler ultrasound examination of the testicles before and after treatment. After euthanasia, the epididymis, testes, liver, and kidney were collected for histological examination. For morphometry of the testis, tubular diameters and seminiferous epithelium height were measured. Sperm concentration and sperm motilities were assessed. Differences among the groups were assessed using the Kruskal–Wallis and Dunn's *post-hoc* test.

**Results:** In all the groups, there were no significant changes in testicular morphology before and after treatment. Histological assessment of the testes showed no alterations in control, no significant alterations in Maca, mild to moderate alterations in THC, and mild alterations in THC + Maca groups. Histological examination of the other organs showed no significant differences among the groups. Tubular diameter showed significantly increased thickening for THC and THC + Maca compared with that for Maca and control. Moreover, seminiferous epithelium height decreased for THC compared with that in the control, Maca, and THC + Maca groups. No statistically significant reduction in the spermatogenic index was observed for THC compared with that for Maca and THC + Maca. Epididymal cross-sections of the groups showed no significant alterations. Sperm concentration and motility were higher for control and THC + Maca groups than in group THC and Maca.

**Conclusion:** *In vivo* maca administration reduced the deleterious effect of THC on testicular parenchyma and semen production.

## Introduction

The medical properties of marijuana and cannabinoids have been widely recognized (1, 2). *Cannabis*-based medicines have proven useful in alleviating autoimmune disorders such as multiple sclerosis, rheumatoid arthritis, and other inflammatory diseases and also play an important role in the treatment of certain neurological diseases such as Alzheimer's disease and amyotrophic sclerosis lateral (2–5). More recent research has shown the ability of *Cannabis*-based medicine to reduce the spread of neoplastic cells (4). These cannabinoids have a high safety profile in relation to the risks of acute toxicity, but not in chronic use (4). Chronic toxic effects have been recognized in reproductive performance (6, 7), and it has been scientifically demonstrated that *Cannabis sativa* and Ruta Graveolens induce hypofertility (8). Currently, the illegal use of *Cannabis sativa* and cannabinoids is widespread and growing, especially in individuals of reproductive age, for recreational, social, medical, and spiritual reasons. Simultaneously, the problems of couple hypofertility is increasing, one-third of which are due to male factors (9). Numerous studies have shown the negative effect of daily marijuana intake on male fertility in both laboratory animals (10) and humans (7).

The direct effect of prolonged exposure to cannabinoids on reproductive organs in various animal species, which interferes with the normal anatomy, histology, and function of male reproductive organs, has been evaluated. Few studies have examined the physical (morphological and histological) effects of the use of exogenous cannabinoids on reproductive organs in humans. Although Kolodny et al. (11) concluded that the chronic use of marijuana in humans does not induce changes in testicular size and histological features of the testicles themselves, numerous studies have shown the opposite. Since endocannabinoid receptor endocannabinoid signaling system (ECSs) are involved in the regulation of the male reproductive system, numerous studies have been conducted to evaluate the effect of cannabinoids on various quality parameters of the semen (7–11). The correlation between cannabinoid exposure and sperm morphological alterations has been poorly studied (7); only one study has shown that this exposure represents a risk factor for the decay of the morphological characteristics of sperm (12).

Both in humans and animals, regular exposure to *Cannabis* induces reduction in sperm concentration (SC) in the ejaculate. Furthermore, the reduction in the number of spermatozoa per ejaculate is dose-dependent (7, 13, 14).

Finally, the literature clearly demonstrates the correlation between exposure to cannabinoids and motility and sperm vitality, both *in vivo* in men and animals and *in vitro*.

Therefore, exogenous cannabinoids, which disturb the physiological homeostasis of ECS receptors, induce harmful energy-dependent effects capable of affecting potential sperm fertility (7, 13, 14).

It is widely documented that oxidative stress plays an important role in the development of hypofertility. A recent study in rats showed that the administration of antioxidants such as melatonin and vitamin C together with the intake of cannabinoids reduces the spermiotoxic effect of the latter (15).

Among the various phytotherapeutics, a tuber, *Lepidium meyenii*, known in common parlance as maca, is recognized by the Andean people and used by the Inca people for its antioxidant power and ability to improve both male and female reproductive functions (16). The aphrodisiac effect of maca as well as its power to increase the reproductive capacity of those who consume it have been scientifically verified (7, 17). Maca has been consumed in Peru for 400 years, both as food and as a medicine. In fact, it has found use in the treatment of rheumatism, respiratory problems, and hormonal imbalances, in the stimulation of metabolism and memory, as a laxative, and finally for the treatment of depression, anemia, leukemia, aids, cancer, alcoholism, and reproduction (18, 19). Other studies have shown that maca can improve the quality of Stallone seed and its refrigerability (9, 20). In addition, maca counteracts the spermiotoxic effects induced by lead acetate in rats (21). The scientific recognition of its properties has led scientists and clinicians to officially include maca as a drug for the treatment of human male hypofertility (7). The aim of this study was to explore *in vivo* the effects of Δ-9-tetrahydrocannabinol (THC) in inducing morphological and histological changes in mouse testes, evaluate sperm motility and concentration, and explore the use of maca in mitigating or boosting the *in vivo* effect of cannabinoids in mice fertility.

## Materials and Methods

### Animal Procedures

The animal protocols used in this work were evaluated and approved by the Animal Use and Ethical Committee (OPBA) of CEINGE, Biotecnologie Avanzate s.c.a.r.l. (Naples, Italy) and by the Italian Ministery of Health [number of authorization 659 del 31.08.17, in accordance with FELASA guidelines and the guidelines defined by the European Communities Council Directive (2010/63/EU)]. Twenty-four C57BL/6 male mice at 6 weeks of age were purchased from Charles River Laboratories International, Inc. and were allowed to acclimate for 2 weeks before the experiments. Mice were divided into four groups: control group (six mice) without any treatment, the first group (nine mice) received 10 mg/kg di Δ^9^-THC in 0.1 ml of sesame oil subcutaneously for 30 days; the second group (10 mice) received 50 mg/kg maca *via* oral administration for 30 days, and the third group (5 mice) received 10 mg/kg di Δ^9^-THC subcutaneously and 50 mg/kg maca by oral administration.

### High-Frequency Ultrasound

High-frequency ultrasound equipment (Vevo 2100, VisualSonics Inc., Toronto, Ontario, Canada) with a multifrequency (30–50 MHz) probe (MicroScan™ MS550D, VisualSonics Inc., Toronto, Ontario, Canada) was used in all procedures.

Mice were divided into three groups based on the established treatment and subjected to ultrasound examination of the testicles before and after treatment. Ultrasound examination was performed under general anesthesia with isoflurane in oxygen (induction phase: 5% isoflurane in 2 L/min oxygen; maintenance phase: 2% isoflurane in 2 L/min oxygen). All ultrasound examinations were performed before treatment and one day after treatment.

Each examined animal was placed in a dorsal decubitus position on the handling table of the Vevo imaging station (Vevo Integrated Rail System III; VisualSonics Inc., Toronto, Ontario, Canada), and vital signs (temperature, heart rate, and respiratory rate) were recorded using a dedicated monitoring system. Body temperature was maintained at 36 ± 5°C *via* an infrared lamp. After positioning, the animal was tricotomized in the pubic and abdominal regions. Each ultrasound session lasted ~45 min. For each testicle, the mediolateral, dorsoventral, and craniocaudal diameters were measured. The volume (mm^3^) of each testicle was calculated using the ellipsoid formula (width × depth × length × xπ/6). Thereafter, a 3D acquisition of mouse testes was performed: a set of consecutive 2D image planes of the testicles were acquired and then automatically reconstructed into 3D views.

Vascularization of tissues within the testicles was assessed using 2D and 3D color-Doppler (36.1 mm/s velocity, 25 dB Doppler gain), and a percent vascularity value (PV%) was provided after the volume had been created (mm^3^). The PV% provides the percentage of the volume that contains flow detected from the color Doppler image. All ultrasonographic assessments were performed by the same trained physician (S.A.), who was unaware of the results obtained in the previous evaluation and blinded to the mice group and pathological results.

### Histopathology and Morphometry

After treatment and the last ultrasound examination, mice were euthanized with overdose of Isoflurane: Isoflurane (Iso-vet®, 1,000 mg/ml, EDRA S.p.A., Italy) were delivered *via* a custom fitted anesthetic machine (Vet-Equipe, Inc., Livermore, CA, USA) that allowed the direct introduction of the gas into the anesthetic chamber. Afterwards mice were subjected to cervical dislocation according to the European rules about animal experimentation.

The testes, liver, kidney, and colon were harvested and preserved in 10% neutral buffered formalin (code no. 05-01007Q, Bio-Optica, Milan, Italy), dehydrated, and embedded in paraffin (code no. 06-7920, Bio-Optica, Milan, Italy). Paraffin blocks were cut into 4-μm-thick sections and stained with hematoxylin and eosin for analysis of morphology.

For the liver, kidney, and colon histologic assessment, several parameters were semiquantitatively evaluated separately by two independent, experienced pathologists (O.P. and D.DB.) in a blinded fashion, with good concordance (Cohen's κ = 0.913, *P* < 0.001).

For the liver histological examination, three main broad categories of histological features were analyzed: steatosis, inflammation, and necrosis. The grading system was adapted from Kleiner et al. (22), as previously described (23). Kleiner's grading system considers the following histological variables: severity of steatosis (quantified by the evaluation of parenchymal involvement by steatosis): score 0, <5%; score 1, 5–33%; score 2, >33–66%, score 3, >66%; location (predominant distribution pattern): zone 3, score 0; zone 1, score 1; azonal, score 2; inflammation: lobular inflammation (overall assessment of all inflammatory foci): score 0, no foci; score 1, <2 foci per ×200 magnification field; score 2, 2–4 foci per ×200 magnification field; score 3, >4 foci per ×200 magnification field; necrosis: score 0, present; score 1, absent.

For the kidney, the examined histologic features were: (1) epithelial degeneration, (2) glomerular atrophy, (3) vascular changes, (4) stromal fibrosis, and (5) tubular atrophy. When present, the damage was evaluated semiquantitatively as 0: none, 1: mild, 2: moderate, or 3: severe (24).

For the colon, the histologic scoring system was adapted from Coretti et al. (25) as follows: (a) the severity of inflammatory cell infiltration was evaluated based on the percentage of leukocyte density in the lamina propria area and estimated in a high-power field representative of the section (0 for no signs of inflammation, 1 for minimal <10%, 2 for mild 10–25% with scattered neutrophils, 3 for moderate 26–50%, 4 for marked >51% with dense infiltrate); (b) The extent of the inflammation was estimated as expansion of leukocyte infiltration (0 for none, 1 for mucosal, 2 for mucosal and submucosal, and 3 for mucosal, submucosal, and transmural levels).

Morphometry of the testis was carried out as previously described by other authors (26, 27), with modifications. Micrographs of experimental and control animals were acquired under a light microscope (Nikon Eclipse E600) attached to a microphotography system (Nikon digital camera DMX1200). For morphometric analysis, setting scale and conversion of values from pixels to micrometers were obtained from a picture with known distance in micrometer. Transverse sections of testes with at least 20 round or nearly round seminiferous tubules were chosen randomly to measure tubular diameters and seminiferous epithelium height for each animal regardless of the stage of the seminiferous epithelium cycle (26) using images obtained at ×100 magnification. The diameter (D) of the seminiferous tubules was measured across the minor and major axes of the tubules by calculating the average of two diameters, D1 and D2. The same tissue section used for measuring tubular diameters was used to measure the seminiferous epithelium height. For this analysis, two perpendicular lines in each field were drawn from the basement membrane (tunica propria) to the tubule lumen (luminal border). The mean of these two values was considered as the height of the seminiferous tubule.

For tubular spermatogenesis index evaluation and quantification, we applied a ten-point scoring system formulated by Johnsen (28) and used both in human and experimental pathology because of its good reproducibility (29). The Johnsen criteria were established according to the profile of the cells encountered along the seminiferous tubules, ranging from no cells to complete spermatogenesis.

### Semen Collection and Evaluation

Immediately after euthanasia, the cauda epididymis and the vasa deferentia were excised. The tissues were incised and placed into a 2-ml Eppendorf with 500 μL of pre-warmed Dulbecco's phosphate-buffered saline solution (Sigma-Aldrich, Milan, Italy). Spermatozoa were allowed to swim up into the medium for at least 30 min at 35°C.

SC was determined using a Bürker chamber at phase contrast (400 × magnification), and the results are presented in sperm cells/mL. Sperm motility was evaluated by placing 10 μL of pre-warmed (37°C) semen suspension between a pre-warmed slide and a coverslip. The slides were examined for total motility (%), as well as rapid and slow progressive motile sperm (%) by a blinded investigator using a phase contrast microscope (Leitz Laborlux K Microscope, Leitz, Italy) at 100 × magnification and heating stage (37°C). For each sample, 10 different randomly selected fields were evaluated.

Numerical variables are reported as medians with interquartile ranges (25th, 75 percentile). Differences among groups were assessed using the Kruskal–Wallis test, followed by Dunn's *post-hoc* test. Statistical significance was set at *p* < 0.05. All analyses were conducted using the statistical platform R (ver. 4.0.1).

## Results

**Figure 1:**
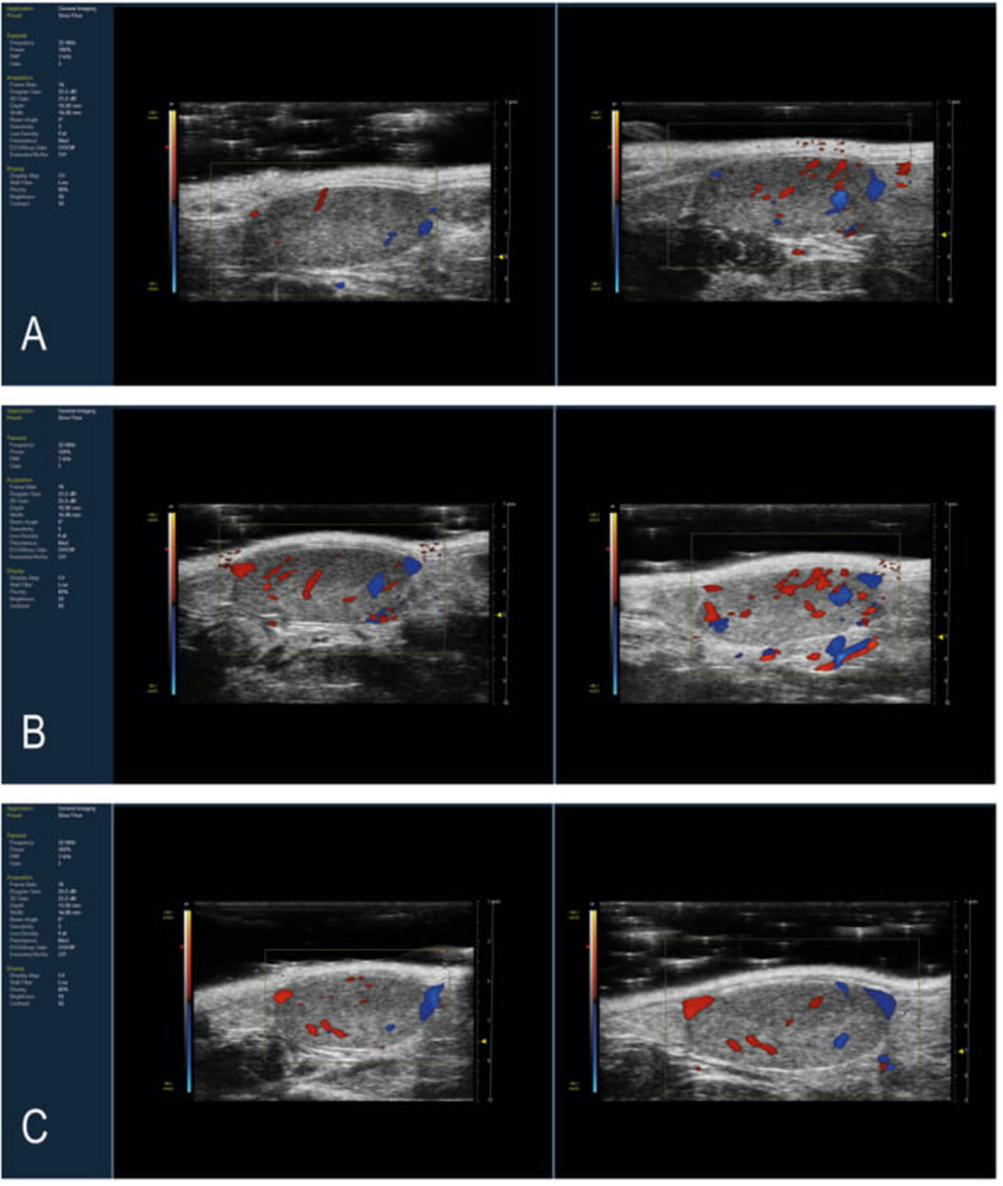
Representative Longitudinal Scan of mouse testis with color Doppler HFUS image. Images of pre-treatment and post-treatment mice testis with **(A)** 10 mg/kg THC, **(B)** 50 mg/Kg maca, and **(C)** 10 mg/kg Δ^9^-THC and 50 mg/Kg maca. After 30 days of treatment, the THC group showed more intense vascularization than the baseline. In contrast, the Maca and Maca + THC groups showed less differences in pre- and post-treatment vascularization.

B-mode acquisition in the transverse and longitudinal planes, followed by a motor 3D-B-mode and -Color Doppler Mode reconstruction of both testicles was performed in all 24 mice before and after treatment (Figure 1). Mice imaged before treatment were considered as controls.

In all the groups, there were no significant changes in testicular morphology before and after treatment, nor in the echogenic structures evaluated. The testicles had a testicular volume ranging from 58.157 to 84.205 mm^3^ pre-treatment and a volume of 54.959 to 85.883 mm^3^ post-treatment. No significant differences were evident among the groups and between all mice pre- and post-treatment.

**Figure 2:**
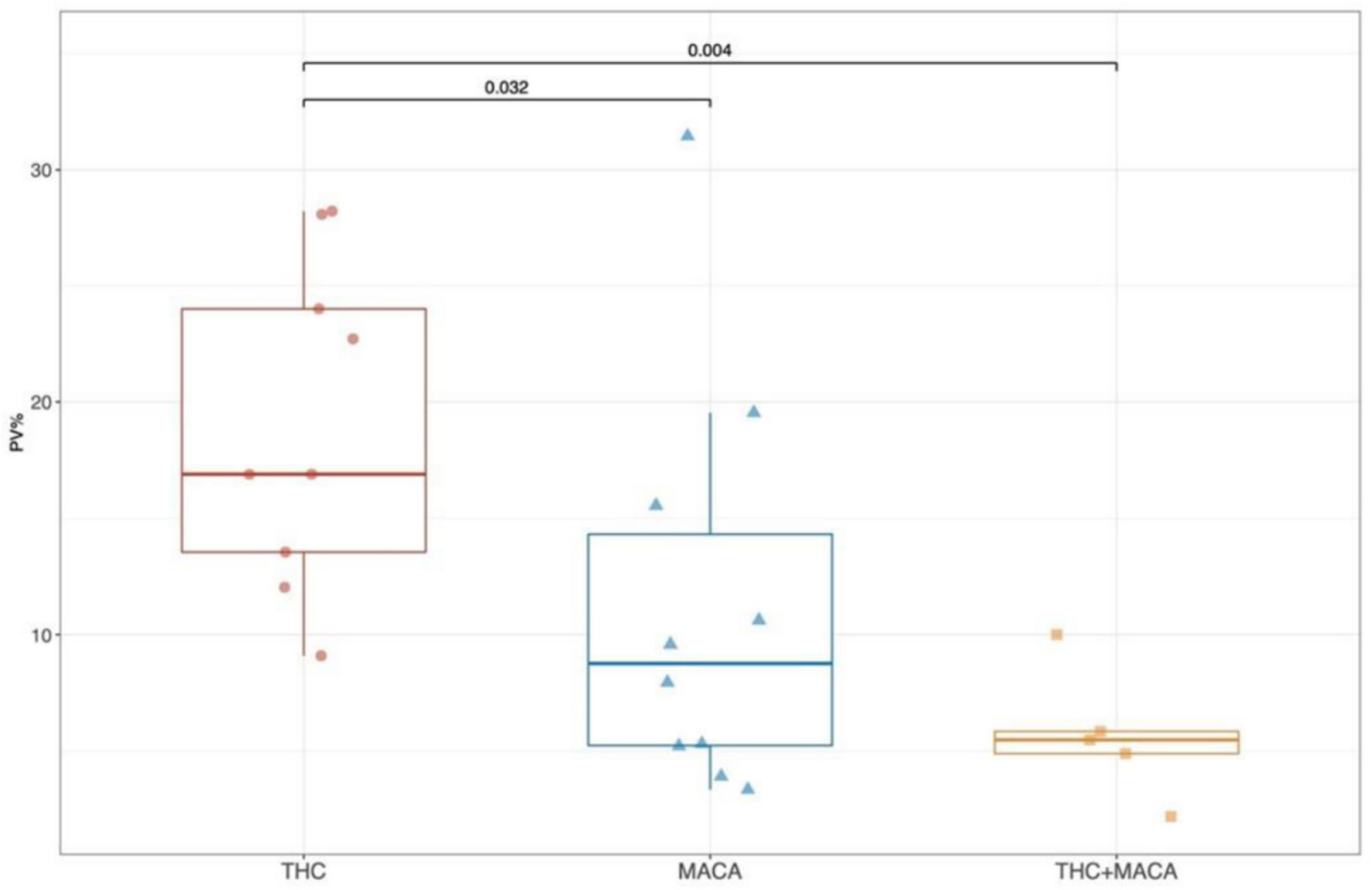
Percentage of vascularization from color Doppler images. Boxplot showing the distribution of post-treatment PV (%) stratified by experimental groups. Boxes represent the 25th to 75th percentile, the line represents the median and the whisker represents the min and max aside outliers that are reported outside the whiskers.

Similar to the tridimensional analysis, we reported the percentage of vascularization (PV%) in all the groups pre- and post-treatment (Figure 2). No significant differences were found among pre-treatment groups (Figure 2). After treatment, the THC group showed a significantly higher PV% than the Maca (*p* = 0.032) and Maca + THC group (*p* = 0.004; Figure 2).

### Histopathology and Morphometry

Morphological analysis was performed for the 24 treated and 4 untreated C57BL/6 mice. Histological examination of formalin-fixed and paraffin-embedded sections of the liver, kidney, and cecum showed no evident histopathological changes for the selected parameters and no statistically significant difference among mice groups.

Histological assessment of testes from mice of the control group showed no alterations with a normal histoarchitecture that consisted of uniform, well-organized seminiferous tubules with complete spermatogenesis and normal interstitial connective tissue. Seminiferous tubules had an intact epithelium with a full complement of spermatogenic cells. Mature spermatozoa filled with tubule lumens and interstitial tissue had a normal distribution of Leydig cells.

**Figure 3:**
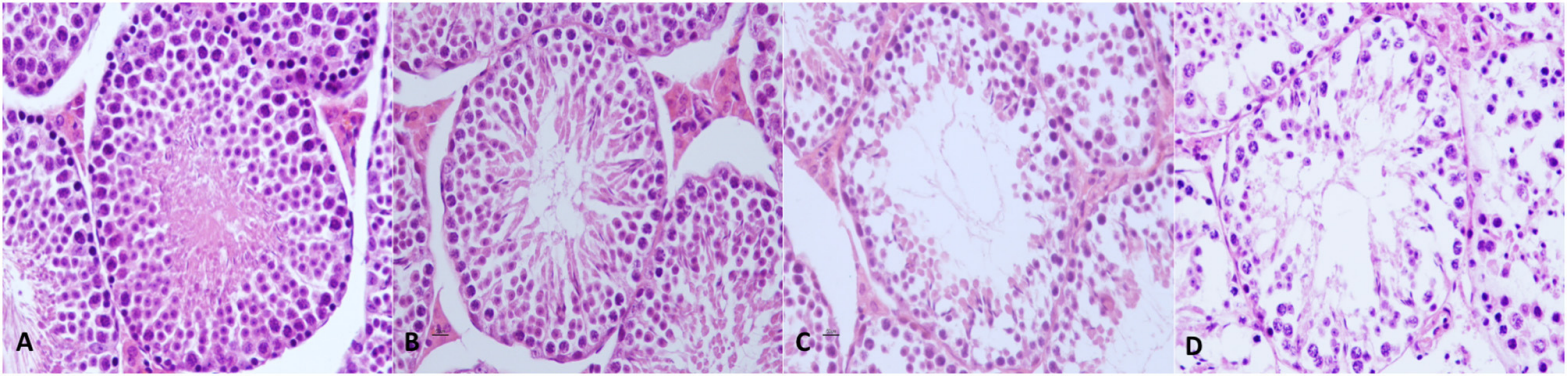
Mouse testis from control and experimental groups. **(A)** Testes from mice of the control group showed normal histoarchitecture with uniform, well-organized seminiferous tubules, and complete spermatogenesis. **(B)** In experimental group 1, no severe and significant alterations were observed in testicular parenchyma nor in spermatogenesis. **(C)** In experimental group 2, transverse sections of the testis showed scattered mild to moderate alterations, which were present mostly in the multifocal detachment of germinal epithelium, irregular and buckled basement membrane, tubular deformation and degeneration, shrunken seminiferous tubules, and increased luminal diameter. **(D)** In experimental group 3, mild alterations such as the detachment of germinal epithelium and reduced population of mature spermatozoa are shown. Hematoxylin and eosin, original magnification 40×.

In the control group and experimental group 3 (THC + Maca), no severe and significant alterations were observed in testicular parenchyma or spermatogenesis. In experimental group 1 (THC), transverse sections of the testis showed mild to moderate pathologic modifications accounting for almost 45% of the testicular parenchyma. Pathologic findings consisted mostly of multifocal detachment of the germinal epithelium, irregular and buckled basement membrane, tubular deformation and degeneration, several shrunken seminiferous tubules, and multifocally increased luminal diameter. In experimental group 2 (Maca), transverse sections of the testis showed an overall normal histoarchitecture of the testicular parenchyma with scattered seminiferous tubules lined by intact epithelium and normal spermatogenesis. A small number of seminiferous tubules, accounting for ~25% of the testicular parenchyma, showed mild alterations such as detachment of the germinal epithelium and a reduced population of mature spermatozoa. Representative pictures of testicular morphology in the control and experimental groups are shown in Figures 3A–D.

Morphometric results are summarized in Table 1. Morphometric measurements showed that tubular diameter significantly decreased in experimental groups 1 and 2 compared with control group and experimental group 3 (*p* < 0.05). Moreover, seminiferous epithelium height decreased significantly in experimental group 1 compared with control group and experimental groups 2 and 3 (*p* < 0.01). The spermatogenic index had a level of 10 (complete spermatogenesis with many spermatozoa) in the control group and experimental group 3, but shifted from 10 to 9 (many spermatozoa present, but germinal epithelium disorganized with marked sloughing or obliteration of lumen) in experimental groups 1 and 2. Therefore, a slight but not statistically significant reduction in the spermatogenic index was observed in experimental group 1 (*p* < 0.001) compared with experimental groups 2 and 3.

**Table 1:** Morphometry of the testis of control and experimental group 1 (THC), group 2 (Maca), and group 3 (THC + Maca).

**Parameter**	**Control**	**Group 1 (THC)**	**Group 2 (Maca)**	**Group 3 (THC + Maca)**
Seminiferous tubular diameter	215.05, 27.6^a^	167.8, 27.4^b^	178.8, 15.2^b^	209.3, 29.5^a^
Seminiferous epithelial height	72.35, 9.87^a^	43.16, 0.69^b^	59.8, 2.7^a^	66.15, 3.4^a^

*Different subscript letters indicate significant differences between groups (p <0.05)*.

Epididymal cross-sections of control groups, as well as experimental groups 1, 2, and 3, showed no significant alterations. The epididymal lumen was filled with spermatozoa, and the epithelium showed an intact basement membrane, epididymal tubules, pseudostratified columnar epithelium, and interstitial areas.

### Semen Parameters

**Figure 4:**
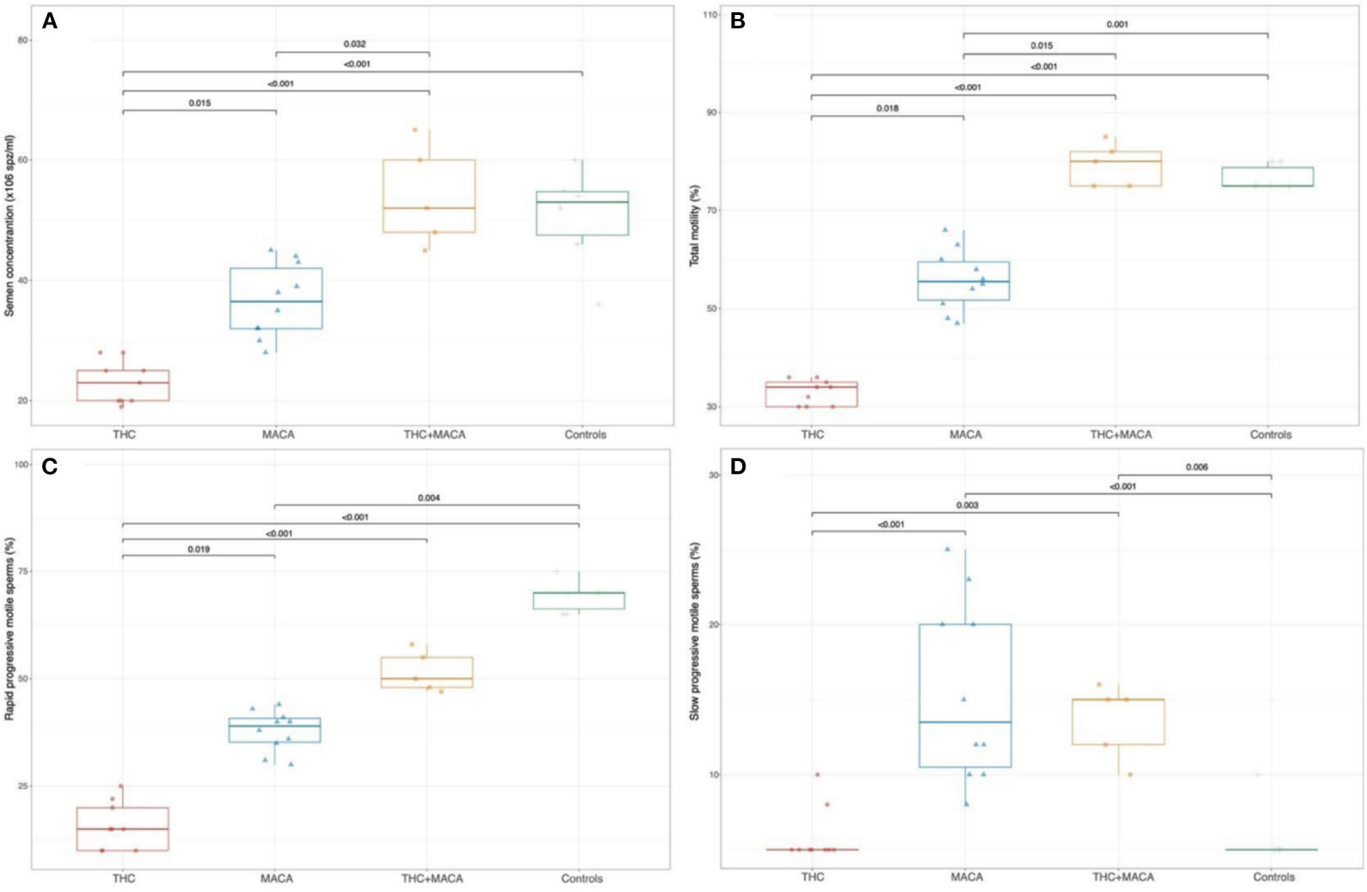
Semen parameters in control and experimental groups. Boxplot showing the distribution of semen parameters stratified by experimental groups. Semen concentration **(A)**, total motility **(B)**, the percentage of rapid and slow progressive motile sperm **(C,D)**. Boxes represent the 25th−75th percentile, the line represents the median and the whisker represents the min and max aside outliers that are reported outside the whiskers.

Semen evaluation was performed on the 24 treated mice and in 6 untreated c57/BL/6 mice used as controls. Significant differences in all semen-related variables were found among the groups (Figure 4). The THC group showed a significantly lower semen concentration (23 [20; 26.5] × 10^6^ spz/ml) than the Maca group (36.5 [31.5; 43.2] × 10^6^ spz/ml; p = 0.015), THC + Maca (52 [46.5; 62.5] × 10^6^ spz/ml; *p* < 0.01) and control groups (53 [43.5; 56.2] × 10^6^ spz/ml; *p* < 0.001). Maca administration resulted in lower semen concentrations in the THC + Maca group (*p* = 0.032).

Total motility was significantly reduced in the THC group [34 (30; 35.5)] and Maca group [55.5 (50.2; 60.8)] compared with that in the control group (75 [75; 80]; *p* < 0.001 and *p* = 0.036, respectively). The THC group also showed a significantly reduced total motility compared to the Maca group (*p* = 0.018) and THC + Maca group (80 [75; 83.5]; *p* < 0.001).

The percentage of rapid progressive motile sperms was significantly reduced in the THC group (15 (10, 23)) and Maca group (39 [34; 41.5]) than in the control group (70 [65; 71.2]; *p* < 0.001 and *p* = 0.004, respectively). The THC group showed a significantly reduced percentage of rapid progressive motile sperm with respect to the Maca (*p* = 0.019) and THC + Maca group (50 [47.5; 56.5]; *p* < 0.001).

With respect to slow progressive motile sperms (%), both the control (5 [5; 6.2]) and THC groups (5 [5; 6.5]) showed significantly reduced percentages when compared with the Maca (13.5 [10; 20.8]; *p* < 0.001 for both) and THC + Maca (15 [11; 15.5]) groups (*p* = 0.006 and 0.003, respectively).

## Discussion

Studies on human reproduction are challenging, given ethical considerations, and the results on the impact of marijuana are confounded by socioeconomic factors and drug variability (9). Strong efforts have been made during the years to elucidate the effect of marijuana on reproduction in human and animal models (9, 30). One of the purposes of this study was to verify the effect of THC administration *in vivo* on the characteristics of epidydimal mouse sperm cells.

Furthermore, studies in humans have suggested that dietary supplementation with antioxidants reduces seminal oxidative stress and improves semen quality, particularly in subfertile males (9, 31–33). Many studies have focused on the use of natural antioxidants from terrestrial plants to prevent sperm damage caused by reactive oxygen species (ROS) (34–36). Maca is a traditional Andean crop used as a nutraceutical for the fertility-enhancing properties associated with its antioxidant activity (37, 38). Moreover, horses with maca dietary supplementation showed an improvement in semen quality during cooling by protecting testicular cell membranes and mitochondria from oxidative stress (9). To date, the *in vivo* effects of THC combined with maca have not been explored. Ultrasound examination is the imaging technique of choice to explore *in vivo* morphology and vascularization of soft tissue (39), including the testis, in experimental animal models of disease (7, 40).

In our study, *in vivo* ultrasound examination showed the absence of grossly morphologic alterations in mice treated with THC, maca, and the combination of the two treatments. However, we found a significant improvement in the percentage of vascularization in mice treated with THC. This could be attributed to the vascular congestion in the seminiferous tubules of testes, also reported by others (41). We speculate that the administration of THC in animal models leads to an early stage of vascular congestion and subsequently, to vascular damage, especially for a prolonged administration of up to 6 months. However, the increase in the percentage of vascularization by itself can induce hyperthermia and consecutively hypofertility. The PV % decreases when mice are treated with maca, and this is even more evident when mice are treated with THC and maca. Maca is confirmed to have a beneficial effect related to the decrease in oxidative stress, which could explain the improvement of the fertility of mice treated with both maca and THC. Finally, maca used alone did not demonstrate the same effect in improving vascularization *in vivo* (42).

Histological evaluations was performed in order to identify possible alterations subsequent to the oral administration of THC and/or maca. The analysis of the selected organs other than testis did not reveal any abnormalities. The effect of THC and Maca on spermatogenesis were evaluated by morphometric parameters and morphological evaluation of testis histology using tubular spermatogenesis index, already reported in literature (26, 27). Those analysis revealed very mild to moderate alterations in parenchymal cytoarchitecture and spermatogenesis in experimental groups compared to the controls. The administration of THC affected the spermatogenesis mostly at the stage of spermiation, showing detachment of the germinal epithelium, exfoliation of spermatocytes, multifocally increased luminal diameter, and a slight reduction in spermatogenesis. However, maca administration seems to reverse the effect of THC on spermatogenesis. Similar results were found after administration of lead acetate plus maca (21).

In line with the morphometric evaluation of seminiferous tubules, the *in vitro* semen evaluation showed a drastic reduction in semen concentration and a loss of sperm motility, confirming the negative effect of THC on male fertility. Despite a large number of recent studies, the results of whether THC affects the ability of sperm to fertilize and generate embryos remains unclear, and the effects of cannabinoids are controversial. The association between the chronic use of THC and abnormalities in sperm count, concentration, motility, and morphology, as well as structural changes in the testis in humans, has been widely reported and reviewed in the literature (9, 43, 44). However, a recent study conducted in male mice showed opposite results, with no negative effect of THC on the male reproduction process (45). Furthermore, the reduction in motility and ATP in sperm treated with THC was dose-dependent (45–48). The mechanism by which THC induces sperm damage is still under investigation. THC activates cannabinoid receptors, which are part of the endogenous endocannabinoid system. This system is a relatively novel system located in the hypothalamus, pituitary, and gonads in both sexes and is involved in spermatogenesis and sperm function (44, 49). The negative effects of THC on testicular morphology and spermatogenesis may depend on the modulation of cannabinoid receptors that are present on Sertoli and Leydig cells and that modulate the balance of molecular signaling and nurturing the microenvironment (50). Modulation of cannabinoid receptors such as CB1 (on Leydig cells) and CB2 (on Sertoli cells) have been suggested to induce local reduction of testosterone production and apoptosis of Sertoli cells, respectively, hence affecting sperm development (51, 52). In the last few years, a growing amount of data has underlined the potential role of oxidative stress in the mechanism of action of THC (53, 54). The risk of stroke in young *Cannabis* users has recently been correlated with the generation of reactive ROS, leading to oxidative stress (53). Moreover, a recent study conducted *in vivo* on rats showed that THC induced cerebral mitochondrial dysfunction and increased hydrogen peroxide production (54). Since oxidative stress is involved in male infertility, different studies have examined the role of this stress in *Cannabis*-associated sperm alterations (55, 56). These studies confirmed the implication of oxidative stress in *Cannabis*-induced spermatotoxicity (55, 56). In our study, oral administration of maca (group 2) and the combination of THC and maca (group 3) interestingly showed little to no pathologic effect on testis and spermatogenesis. However, these data were not completely corroborated by the *in vitro* evaluation of semen, which revealed a harmful effect of maca on SC and sperm motility. Indeed, other investigators have observed a beneficial effect of maca administration on spermatogenesis in mice, improving sperm count and motility (7, 9, 16, 57). Meanwhile, the use of only maca reduced sperm motility and concentration, and the supplementation of mice receiving THC with maca improved sperm characteristics.

Based on results, authors can hypothesize different mechanisms used by Maca to reverse the deleterious effect of THC. Since mice treated with THC plus Maca have similar histological results to control group, maca could protect the testis from spermatogenic disruption caused by THC preventing apoptosis of the developing germ cells and improving the number of cells progress through the spermatogenesis. Previous study suggests that Maca reversed deleterious effects due lead acetate on spermatogenesis by protecting onset of mitosis and spermiation (21).

Positive effect of Maca is correlated with its antioxidant effect that reduces THC-associated sperm damage caused by oxidative stress. Similar ameliorative effects in *Cannabis sativa*-associated spermiotoxicity were reported with the use of other antioxidants, such as a combination of melatonin and vitamin C (15, 55). The negative effect of the administration of maca on semen can be due to an alteration of the endogenous antioxidant systems by this antioxidant. Oxidative stress caused by ROS is physiologically balanced by endogenous antioxidant systems. The authors' hypothesis is that in these mice, under physiological conditions, antioxidant supplementation is not necessary because the balance between pro-oxidants and antioxidants is already in place for the natural evolution of sperm physiology. The addition of antioxidants in the diet or in the semen-targeted improvement of semen production and quality should take into account the endogenous production of antioxidants, which varies greatly between individuals (9, 58, 59). There is a limitation of the study and potential bias caused by the subjective evaluation of sperm motility.

In conclusion, this study confirmed that the oral administration of maca prevents the harmful effect of THC on mouse spermatogenesis and spermatozoa features, and it lends further credibility to the hypothesis that Maca could be an alternative treatment for male infertility. In order to evaluate the biological activity of maca during oral supplementation, successive studies should be carried out on redox status measurements and reproductive hormonal modifications in treated mice.

## Data Availability Statement

The original contributions presented in the study are included in the article/supplementary material, further inquiries can be directed to the corresponding author/s.

## Ethics Statement

The animal study was reviewed and approved by Animal Use and Ethical Committee (OPBA) of CEINGE, Biotecnologie Avanzate s.c.a.r.l. (Na-ples, Italy) and by the Italian Ministry of Health [number of authorization 659 del 31.08.17, in accordance with FELASA guidelines and the guidelines defined by the European Communities Council Directive (2010/63/EU)].

## Author Contributions

All authors listed have made a substantial, direct, and intellectual contribution to the work and approved it for publication.

## Funding

This study was supported by a grant from the Department of Veterinary Medicine and Animal Productions University of Naples Federico II, Italy, in partnership with the Interdepartmental Center of Veterinary Radiology, University of Naples Federico II, and CEINGE, Advanced Biotechnology, University of Naples Federico II.

## Conflict of Interest

The authors declare that the research was conducted in the absence of any commercial or financial relationships that could be construed as a potential conflict of interest.

## Publisher's Note

All claims expressed in this article are solely those of the authors and do not necessarily represent those of their affiliated organizations, or those of the publisher, the editors and the reviewers. Any product that may be evaluated in this article, or claim that may be made by its manufacturer, is not guaranteed or endorsed by the publisher.
